# Thermogelling Behaviors of Aqueous Poly(N-Isopropylacrylamide-co-2-Hydroxyethyl Methacrylate) Microgel–Silica Nanoparticle Composite Dispersions

**DOI:** 10.3390/ma14051212

**Published:** 2021-03-04

**Authors:** Byung Soo Hwang, Jong Sik Kim, Ju Min Kim, Tae Soup Shim

**Affiliations:** 1 Department of Chemical Engineering, Ajou University, Suwon 16499, Korea; brianify@ajou.ac.kr; 2 Department of Energy Systems Research, Ajou University, Suwon 16499, Korea; kjsik@ajou.ac.kr

**Keywords:** stimuli-responsive hydrogels, thermogelling polymers, sol–gel transition behaviors, complex colloidal systems

## Abstract

Gelation behaviors of hydrogels have provided an outlook for the development of stimuli-responsive functional materials. Of these materials, the thermogelling behavior of poly(N-isopropylacrylamide) (p(NiPAm))-based microgels exhibits a unique, reverse sol–gel transition by bulk aggregation of microgels at the lower critical solution temperature (LCST). Despite its unique phase transition behaviors, the application of this material has been largely limited to the biomedical field, and the bulk gelation behavior of microgels in the presence of colloidal additives is still open for scrutinization. Here, we provide an in-depth investigation of the unique thermogelling behaviors of p(NiPAm)-based microgels through poly(N-isopropylacrylamide-co-2-hydroxyethyl methacrylate) microgel (p(NiPAm-co-HEMA))–silica nanoparticle composite to expand the application possibilities of the microgel system. Thermogelling behaviors of p(NiPAm-co-HEMA) microgel with different molar ratios of N-isopropylacrylamide (NiPAm) and 2-hydroxyethyl methacrylate (HEMA), their colloidal stability under various microgel concentrations, and the ionic strength of these aqueous solutions were investigated. In addition, sol–gel transition behaviors of various p(NiPAm-co-HEMA) microgel systems were compared by analyzing their rheological properties. Finally, we incorporated silica nanoparticles to the microgel system and investigated the thermogelling behaviors of the microgel–nanoparticle composite system. The composite system exhibited consistent thermogelling behaviors in moderate conditions, which was confirmed by an optical microscope. The composite demonstrated enhanced mechanical strength at gel state, which was confirmed by analyzing rheological properties.

## 1. Introduction

Phase transition behaviors of colloidal dispersion have long been studied to understand the fundamentals of colloidal interactions and to apply the system to various fields such as cosmetics, pharmaceutics, food industries, paints, inks, slurries, etc. Especially, in-depth investigation of highly complex systems of slurries and pastes based on the knowledge of rheological properties of homogeneous or heterogeneous colloidal formulations paves a promising outlook for the energy industry [1,2]. However, most of the systems applied in the industry are based on hard-sphere-like colloids such as carbon, polystyrene, and silica. In comparison, investigations on soft materials such as hydrogels in the previously discussed fields have had a weaker standing.

Hydrogels are water-absorbing polymers well-known for their bio-compatibility and stimuli-responsivity. The reversible volume phase transition and elastic behaviors of hydrogel have enabled the design of smart materials such as temperature-responsive drug delivery and wound healing materials [3,4,5], remotely controlled soft actuators [6,7], stimuli-responsive plasmonic materials [8], flexible sensors [9,10], etc. In addition, a sparse polymer network in an aqueous medium can be used as a matrix to incorporate nanoparticles [11] and microreactors [12,13]. However, in the past, their intrinsic drawbacks such as weak mechanical strength and mandate for the moist environment have limited the field of applications. Recent achievements addressed these issues: the development of double-network hydrogels [14] which enhances the mechanical durability and replacement of the aqueous medium with a non-volatile organic medium that improves the useability in dry environments [15]. Therefore, we can expect a gradual expansion in the application field of hydrogel-based soft colloids to the field areas in which hard-sphere-like colloids are used while maintaining their functionality.

Of the hydrogels, the thermogelling microgel dispersion is one of the intriguing systems that exhibits reversible sol–gel transition behavior [16]. Unlike the conventional physical gels that maintain a gel state below the phase transition temperature, the thermogelling microgel dispersion shows inverse phase transition from sol to gel as the temperature increases. This inverse phase transition is triggered by the hydrophilic and hydrophobic interactions among microgels at the volume phase transition temperature of the material [17]. Poly(n-isopropylacrylamide) p(NiPAm) microgel dispersion is one of the widely known temperature-responsive colloid system. In general, it shows reversible swelling/shrinkage at a lower critical solution temperature (LCST), which is around 32 °C. Above LCST, the hydrophobic moiety becomes dominant and repels water out from the polymer network of the microgel [18]. The p(NiPAm) colloids show a stable dispersion even at a temperature above LCST. When salt is added, however, it forms bulk gels around LCST due to the weak electrostatic repulsion among microgels [19]. Because this gelation does not require additional crosslinking reactions, they have been studied as an in situ gelation material for biomedical applications. Furthermore, copolymerization of p(NiPAm) with various comonomers has been investigated to harness sol–gel transition behaviors. Many endeavors were put to enhance the mechanical strength of the gel while maintaining the sol–gel transition behavior, and many successes have been reported. For example, copolymerization of NiPAm with methylcellulose [20] or acrylic acid [21] showed that the gel’s mechanical strength could be enhanced with varying concentrations. A different enhancement, the maintenance of the gel volume at LCST, also shared some attention. For instance, copolymerization with 2-hydroxyethyl methacrylate (HEMA) extended the sequential delivery of the protein from the gel scaffold, which can be interpreted as an improvement from subsequent shrinkage of the gel after gelation [22]. Despite many studies on understanding gelation and controlling their mechanical properties, the thermogelling behavior of p(NiPAm)-based microgels in the presence of colloidal additives has not been fully understood. To take advantage of the unique gelation behavior in various colloidal systems, it is necessary to understand the effect of colloidal additives. Following the research mentioned, investigating the phase transition behaviors of microgel–nanoparticle composite systems will prove useful to expand the areas of application to more than just the biomedical field.

In this study, we specifically consider thermogelling behaviors of microgels–nanoparticle composite systems consisted of poly(N-isopropylacrylamide-co-2-hydroxyethyl methacrylate) (p(NiPAm-co-HEMA)) microgels and silica nanoparticles. We prepared aqueous p(NiPAm-co-HEMA) microgel dispersions with various NiPAm:HEMA molar ratios through radical polymerization. Then, thermogelling behaviors of the neat p(NiPAm-co-HEMA) microgel dispersions upon varying salt concentrations were studied by a rheometer. Finally, we added silica nanoparticles to the microgel dispersion to make a model composite system to investigate the thermogelling behaviors.

## 2. Materials and Methods

### 2.1. Materials

N-Isopropylacrylamide (NiPAm, TCI, Tokyo, Japan), 2-hydroxyethyl methacrylate (HEMA, Aldrich, St. Louis, MO, USA), N,N’-methylenebis(acrylamide) (MBA, Aldrich, St. Louis, MO, USA), ammonium persulfate (APS, Aldrich, St. Louis, MO, USA), and sodium dodecyl sulfate (SDS, Aldrich, St. Louis, MO, USA) were purchased and used as received.

### 2.2. Synthesis of p(NiPAm-co-HEMA) Microgels

Microgels of the different molar ratios of NiPAm and HEMA were synthesized by free radical precipitation polymerization. The constituent monomers, NiPAm and HEMA, and the crosslinker, MBA, were dissolved accordingly to the specified molar ratio while the amount of the initiator, APS, and the surfactant, SDS, were kept constant. Six batches of p(NiPAm-co-HEMA) were synthesized, namely, 10:0, 9:1, 8:2, 7:3, 6:4, 5:5, denoting the molar ratio between NiPAm and HEMA. The detailed recipe of each batch is summarized in Table 1. The monomers, crosslinker, and surfactant were dissolved in 498 mL of water. The reaction mixture was transferred to a 1 L-size, three-neck, and double-jacket reactor equipped with a condenser, a nitrogen inlet, and a mechanical stirrer. The reaction mixture was stabilized at 70 °C while stirring at 360 rpm under a nitrogen atmosphere for an hour. Afterward, 0.342 g of APS was dissolved in 2 mL of water and added to the mixture to initiate the polymerization. The reaction time for each batch is specified in Table 1. The resultant microgel dispersion was washed with three successive centrifugations to remove unreacted monomers and the surfactant. Finally, the dispersion was freeze-dried to obtain a solid sample for future use.

### 2.3. Rheological Characterization of p(NiPAm-co-HEMA) Microgels

The gelling behavior of the p(NiPAm-co-HEMA) microgels was characterized by small-amplitude oscillatory shear (SAOS) tests. We used a commercial rotational rheometer (AR-G2, TA Instruments, New Castle, DE, USA) equipped with a cone-and-plate geometry (1°; diameter: 60 mm) to perform the linear viscoelasticity characterization. The storage and loss moduli (G′ and G″) were monitored at the fixed frequency of 0.63 (rad/s) with increasing temperature starting from 20 °C with the increment rate at 1 °C/min.

## 3. Results and Discussions

### 3.1. Synthesis and Characterization of p(NiPAm-co-HEMA) Microgels

**Figure 1:**
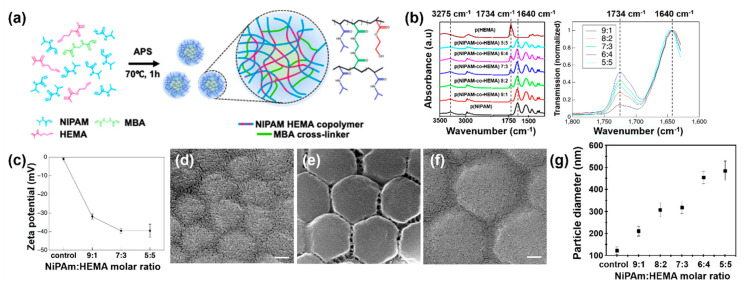
(**a**) Schematic illustration for the synthesis of p(NiPAm-co-HEMA) microgels. (**b**) Fourier-transform infrared spectroscopy (FTIR) data for p(HEMA) polymer, p(NiPAm), and p(NiPAm-co-HEMA) microgels with varying molar ratio between N-isopropylacrylamide (NiPAm) and 2-hydroxyethyl methacrylate (HEMA). For comparison of NiPAm and HEMA signal ratio among samples, normalized FTIR data are plotted for p(NiPAm-co-HEMA) microgels (right). (**c**) Zeta potential of p(NiPAm) and p(NiPAm-co-HEMA) microgels with varying NiPAm:HEMA molar ratio. (**d**–**f**) Scanning electron microscope (SEM) images of p(NiPAm-co-HEMA) microgels with molar ratios between NiPAm and HEMA of (**d**) 9:1, (**e**) 7:3, and (**f**) 5:5, respectively. Scale bar in (**d**–**f**) represents 100 nm. (**g**) Effect of molar composition of HEMA to particle diameter.

The p(NiPAm-co-HEMA) microgels were synthesized by radical precipitation polymerization as illustrated in Figure 1a [23]. We simply mixed two monomers having unsaturated hydrocarbon groups with a crosslinker. Thus, it is assumed that the polymeric structure of the copolymer is random. For Fourier-transform infrared spectroscopy (FTIR) measurement, p(NiPAm-co-HEMA) microgel was freeze-dried to obtain a powdery sample. The FTIR data in Figure 1b confirms the successful synthesis of p(NiPAm-co-HEMA) in accordance with the specified mole ratio as described in the experimental section. The absorption peak at 3275 cm^−1^ and 1640 cm^−1^ are assigned to stretching vibration of the amino group (N–H) and the amide I groups (C=O) in NiPAM, respectively [24]. The peak at 1734 cm^−1^ is assigned to the stretching vibration of the carbonyl group (C=O) in HEMA [17]. As the molar ratio of HEMA increases so does the peak intensity at 1734 cm^−1^, while it dwindles at 1640 cm^−1^. Normalized FTIR data for comparison of NiPAm and HEMA peak ratio of p(NiPAm-co-HEMA) microgels showed an increase of carbonyl group signal as the content of HEMA increased. For the 5:5 sample, however, the intensity of the carbonyl group signal decreased because the microgel was not successfully synthesized. Zeta potential of microgel dispersion with varying NiPAm:HEMA molar ratio was measured at room temperature. For a control sample, p(NiPAm) microgel dispersion, the value of zeta potential was at the isoelectric point with a slightly negative value. It showed a decreasing trend in values as the content of HEMA increased.

The average diameter of p(NiPAm-co-HEMA) microgels shows a degree of swelling. Scanning electron microscope (SEM) images of the freeze-dried p(NiPAm-co-HEMA) microgels in Figure 1d–f indicate uniformly sized, spherical microgels. It was confirmed that the diameter of microgels increased as the composition of HEMA increased, Figure 1g. The increment of particle size is attributed to the good water uptake capability of HEMA. As a result, sparse polymeric networks were formed during the radical polymerization as the amount of HEMA increased, resulting in the formation of larger-, and mechanically weaker microgels. Indeed, when we increased the molar ratio of HEMA to more than 0.5, the structural integrity of p(NIPAM-co-HEMA) microgels could not be maintained. As a result, bulk p(NIPAM-co-HEMA) polymer cakes were observed as gels precipitated. Based on the above results, further studies regarding investigations of colloidal stability and their thermogelling behaviors were mainly focused on the characteristics of p(NiPAm-HEMA) microgel dispersions with NiPAm to HEMA molar ratios of 9:1, 7:3, and 5:5. In addition, further experiments were conducted in neutral pH conditions because colloidal stability under acidic and alkalic conditions was unstable, as shown in Figure S1.

### 3.2. Effect of Salt on the Stability of p(NiPAm-co-HEMA) Microgel Dispersion

**Figure 2:**
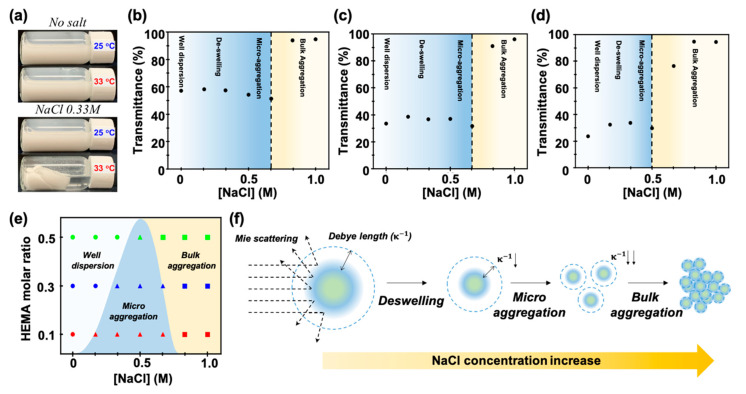
(**a**) Photograph images of thermogelling p(NiPAm-co-HEMA) microgel dispersion upon different temperature conditions. In the absence of salt, the dispersion remained at the sol state for both 25 °C and 33 °C (top panel). In the presence of 0.33 M of sodium chloride (NaCl), microgels dispersion showed volume phase transition from sol to gel state at 33 °C (bottom panel). (**b**–**d**) The transmittance of p(NiPAm-co-HEMA) microgel dispersion with molar ratios between NiPAm and HEMA of (**b**) 9:1, (**c**) 7:3, and (**d**) 5:5, respectively, with respect to NaCl concentration. The bulk aggregation of microgels by the strong ionic strength of solution was expressed in yellow background. (**e**) Summary of dispersion quality of p(NiPAm-co-HEMA) microgels. The microgel showed a well-dispersed (white background) phase, micro aggregation (blue background) phase, and bulk aggregation (yellow background) phase in accordance with NiPAm: HEMA molar ratios and NaCl concentrations. (**f**) Schematic illustration of deswelling of microgel with the increase of the salt concentration.

A notable difference between p(NiPAm-co-HEMA) and p(NiPAm) microgel dispersion is that the former requires the addition of salt and appropriate temperature for successful gelation, Figure 2a. This is because the electrostatic repulsion between the microgels is increased by the hydroxyl group of HEMA. In the presence of HEMA, salt ions are required to screen the electrostatic double layers on the microgels to prompt the formation of a particle network that leads to gelation upon thermal heating. Because adding salt in conventional colloidal dispersion causes destabilization of the system, we first investigated the effect of salt on the stability of p(NiPAm-co-HEMA) microgel dispersions.

The stability of p(NiPAm-co-HEMA) microgel dispersion was investigated by observing the turbidity of the dispersion via a UV–visible spectrometer (SHIMAZU, Kyoto, Japan). 0.04 wt% of p(NiPAm-co-HEMA) microgel dispersion with varying NiPAm to HEMA molar ratios were prepared. As shown in Figure 2b–d, the transmittance of the p(NiPAm-co-HEMA) microgels composed of NiPAm to HEMA molar ratios of 9:1, 7:3, and 5:5 at different NaCl concentrations were measured. The transmittance for each sample without NaCl was measured as 59.7%, 34.7%, and 23.7% for 9:1, 7:3, and 5:5 samples, respectively, which showed a decreasing trend as the amount of HEMA increased. This is because the particle size-dependent Mie scattering was dominant for particles with a size similar to the wavelength of the incident light. In all cases, transmittance was slightly increased at low salt concentration and then started to decrease until it reached the critical salt concentration. Then, a dramatic increase in transmittance was observed. These results can be explained by the change of Mie scattering and Debye length of the system along with the change of the salt concentration. When the salt is added to the system, osmotic pressure applied to the microgel causes the deswelling of the microgels [16,25]. It results in the decrease of the microgel size and thereby decrease of Mie scattering. Therefore, the transmittance of dispersion was increased. As we stated above, an increase of the salt concentration in colloidal dispersion also causes the screening of electric double layer, which results in the decrease of Debye length of microgels. When the Debye length reaches below the critical value for maintaining a stable dispersion, Van der Waals force causes aggregation of microgels followed by sedimentation. Here, a decrease and dramatic increase of transmittance can be explained by microaggregation and bulk aggregation (i.e., sedimentation), respectively. When the dispersion is at the state of microaggregation, the effective size of microgels can be considered to be larger and it results in the decrease of transmittance. Then, a sudden increase of transmittance at bulk aggregation is observed due to the sedimentation. Therefore, a critical salt concentration can be regarded as a point at which bulk aggregation of microgels occurs. The phase behaviors of p(NiPAm-co-HEMA) microgel dispersion with varying NiPAm:HEMA compositions in Figure 2e revealed that the higher the amount of HEMA is, the more the salt concentration-initiated microaggregation occurs. By reflecting on the trends of transmittance for each sample, it is expected that the content of HEMA contributed to the increase of Debye length of microgels, which also agreed with the previous results. The comprehensive behavior of microgel dispersion is schematically described in Figure 2f.

### 3.3. Thermogelling Behaviors of p(NiPAm-co-HEMA) Microgel Dispersion

**Figure 3:**
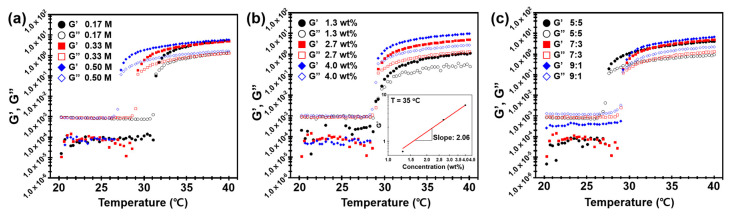
Evolution of storage (G′) and loss (G″) moduli of the p(NiPAm-co-HEMA) microgel dispersion from 20 °C to 40 °C. The procedure was conducted under fixed stress of 0.05 Pa and a frequency of 0.1 Hz (ω= 0.63 rad/s). (**a**) Change in dynamic modulus of the polymer at different salt concentrations. The concentration of polymer and molar ratio between NiPAm and HEMA were kept constant at 2.7 wt% and 7:3, respectively. (**b**) Change in dynamic modulus at different copolymer concentration ($c_{m}$). The concentration of NaCl and molar ratio between NiPAm and HEMA were kept constant at 0.17 M and 7:3, respectively. Log plot between G′ and microgel concentration showed a power-law relationship with a slope of 2.06 (inset). (**c**) Change in dynamic modulus at different molar ratios between NiPAm and HEMA. The concentrations of polymer and NaCl were kept constant at 2.7 wt% and 0.33 M, respectively.

We characterized the thermogelling behaviors of p(NiPAm-co-HEMA) microgel dispersion in accordance with the concentration of NaCl, concentration of microgels and composition of NiPAm and HEMA by the SAOS test results measured with a rotational rheometer (refer to the experimental section for the detailed conditions). Following the conventional method of measuring thermogelling behaviors, the gelation temperature at which the colloidal dispersion changes from sol to gel was determined by observing the cross-over behavior of the storage (G′) and loss (G″) moduli, as shown in Figure 3 [26].

#### 3.3.1. Effect of Salt Concentration

From the prior investigation of the dispersion stability, it can be concluded that the microgels under the speculation are stable up to 0.5 M NaCl. Thus, the effect of salt concentration for thermogelling behaviors was investigated for 2.7 wt% of 7:3 molar ratio of p(NiPAm-co-HEMA) microgel dispersion with NaCl concentrations of 0.17 M, 0.33 M, and 0.5 M. As shown in Figure 3a, larger G″ than G′ was observed initially for all the samples with no significant fluctuation, which proves a liquid-like sol state. As temperature kept increasing, a crossover between G′ and G″ occurred at 31.8 °C, 29.3 °C, and 27.3 °C for samples with salt concentrations of 0.17 M, 0.33 M, and 0.5 M, respectively. The result shows an aggregation of p(NiPAm-co-HEMA) microgels and thereby gelation of the system. Based on the findings, it was concluded that the gelation temperature decreases as the salt concentration increases. Decreasing trends of gelation temperature can be attributed to the screening of electrostatic repulsion by the salt addition, which results in the decrease of Debye length of p(NiPAm-co-HEMA) microgels. In addition, it is notable that the plateau of the G′ and G″ after gelation was formed at a similar magnitude for all the samples. It implies that the mechanical strength of gels in different salt concentrations was not affected.

#### 3.3.2. Effect of Microgel Concentration

Effect of microgel concentration was conducted by comparing 1.3 wt%, 2.7 wt%, and 4.0 wt% of 7:3 molar ratio of p(NiPAm-co-HEMA) microgel dispersion at a fixed NaCl concentration of 0.33 M. As shown in Figure 3b, gelation temperature where the crossover between G′ and G″ occurs did not change by the concentration of p(NiPAm-co-HEMA) microgels. On the other hand, the mechanical strength of gels was affected by the concentration. When the temperature exceeds the gelation temperature, the magnitude of both G′ and G″ at plateau were proportional to the microgel concentration. It is noted that the magnitude of G′ and G″ at sol state (i.e., below gelation temperature) only showed little increases with increasing the concentration of p(NiPAm-co-HEMA) microgels. The current results suggest that the viscous properties (≅G″/ω) at the sol states of the samples are all close to that of pure water, i.e., G″~O(10^−3^) [27], which are expected to be proportional to the volume fractions of the microgel. However, the change in the viscous properties with the increasing microgel concentration is too small to be captured within the sensitivity limit of the rotational rheometer. On the other hand, it is clear that their gel strengths are significantly affected by the formation of denser physical networks between microgels during the gelation process as the microgel concentration increases [26]. The relationship between $c_{m}$ and gel strength (G′) clearly shows a power-law relationship (inset of Figure 3b), which is consistent with the previous studies on the gelation of particulate systems [28]. The power-law exponent is 1 when the particle volume fraction is close to the gelation particle volume fraction ($\phi_{g}$), which increases to 3–5 as the volume fraction significantly deviates from $\phi_{g}$ [28]. Therefore, it can be concluded that the current particle volume fraction ($\approx kc_{m}$) range is not far from $\phi_{g}$ for p(NiPAm-co-HEMA) microgels, in which k is a proportionality constant between the particle concentration and volume fraction.

#### 3.3.3. Effect of NiPAm:HEMA

The effect of NiPAm:HEMA molar ratio in p(NiPAm-co-HEMA) microgel was investigated by preparing 2.7 wt% of p(NiPAm-co-HEMA) microgels that composed of NiPAm and HEMA with the molar ratio of 5:5, 7:3, and 9:1, respectively, at a fixed NaCl concentration of 0.33 M. As shown in Figure 3c, the effect of NiPAm:HEMA molar ratio was somewhat less clear than that of NaCl and microgel concentrations. In both sol and gel states, magnitudes of G′ and G″ were slightly higher with a lower HEMA molar ratio. For gelation temperature, there was a negligible difference between samples with a NiPAm:HEMA molar ratio of 7:3 and 9:1, and a decrease of gelation temperature was observed for the sample with a NiPAm:HEMA molar ratio of 5:5. These results are attributed to the difference in size and thereby the different volume fractions of microgels in the dispersion. As we discussed in Figure 2, the size of microgels was increased due to the good water uptake capability of HEMA. Because every sample was prepared to have the same weight percent of microgels, the volume fraction of microgels will be higher for the larger microgels. In addition, the composition of HEMA can also cause the change in the electric double layer and Debye length of microgels. Considering the aforementioned observations comprehensively, the change in composition affects the gelation properties, but it is not an appropriate variable for precise control of the gelation temperature or mechanical properties of the gels.

### 3.4. Thermogelling Behaviors of p(NiPAm-co-HEMA) Microgel with Silica Nanoparticle Composites

**Figure 4:**
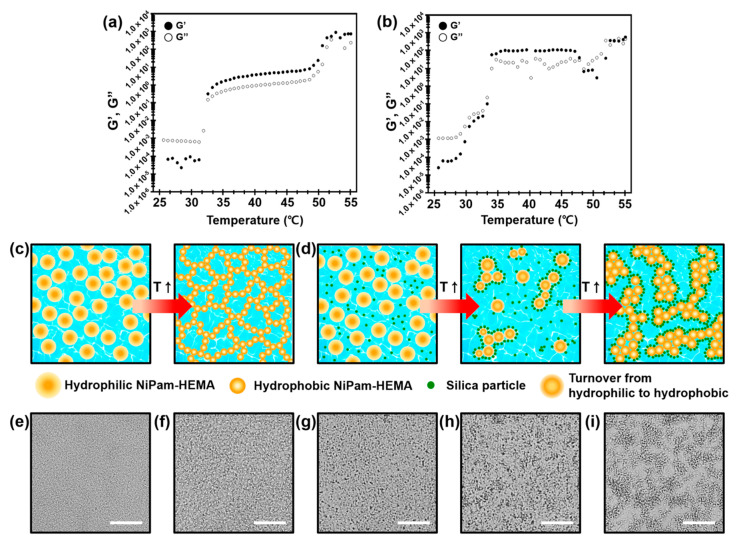
(**a**,**b**) Thermogelling behavior of 2.7 wt% p(NiPAm-co-HEMA) microgels with 7:3 of NiPAm:HEMA molar ratio at 0.17 M of NaCl and silica nanoparticle composite. A comparative study between (**a**) the neat p(NiPAm-co-HEMA) and (**b**) the mixture of the microgel and the silica nanoparticle of 1:1 weight ratio. (**c**,**d**) Schematics of gelation process of (**c**) a neat p(NiPAm-co-HEMA) microgel dispersion and (**d**) the p(NiPAm-co-HEMA) microgel–silica nanoparticle composite. Time-resolved microscopic images showing sol–gel transition of (**e**,**f**) a neat p(NiPAm-co-HEMA) microgels and (**g**–**i**) p(NiPAm-co-HEMA) microgel–silica nanoparticle composite.

Investigation of thermogelling behaviors of p(NiPAm-co-HEMA) microgel in the presence of silica nanoparticles was conducted. Here, p(NiPAm-co-HEMA) microgels having a 7:3 molar ratio between NiPAm and HEMA were mixed with LUDOX silica (Aldrich, St. Louis, MO, USA) nanoparticles with weight ratios of 1:1 and 1:5, respectively. Rheological behaviors of samples showed an increasing trend of elastic modulus upon gelation while moderately maintaining volume phase transition temperature. As shown in Figure 4a,b, the microgel–silica nanoparticle composite gel having a 1:1 weight ratio exhibited an increase of G′ and G″ by 10 folds in comparison to the gel in the absence of silica nanoparticles. This can be attributed to the jammed silica nanoparticles around the p(NiPAm-co-HEMA) microgels during gelation. As illustrated in Figure 4c, volume phase transition of p(NiPAm-co-HEMA) microgels takes place when the surface moiety of microgels change from hydrophilic to hydrophobic. In this case, the hydrophobic nature of microgels cause them to be percolated, which results in the continuous microgel networks forming the bulk gel. In the presence of silica nanoparticles, it is readily adsorbed on the surface of microgels by electrostatic interaction. As a result, percolated microgel network can be reinforced by jammed silica nanoparticles around the microgels, as illustrated in Figure 4d. To investigate the gelation behaviors further, we observed volume phase transition behaviors of microgels through a transmission optical microscope. A neat p(NiPAm-co-HEMA) microgels at sol state showed homogeneous dispersion, as shown in Figure 4e, and it gradually percolated as the temperature reached 60 °C, shown in Figure 4f (see Video S1 for details). In the case of the microgel/silica nanoparticle composite, however, small aggregates were observed at room temperature (Figure 4g), and they locally aggregated as temperature increased (Figure 4h). These behaviors can be explained by electrostatic attractions among positively charged microgels and negatively charged silica nanoparticles. At the elevated temperature of 60 °C, the global aggregation was observed (Figure 4i), in which larger and locally aggregated colloidal grains were prominent (see Video S2 for details). From the results above, it is concluded that the dispersion stability has to be carefully considered for successful gelation of microgel–nanoparticle composite system. Indeed, we found an unstable sol–gel transition behavior when the concentration of silica nanoparticles was increased further. We conducted experiments for the p(NiPAm-co-HEMA) microgels and silica nanoparticle composites having 1:5 weight ratios. Although global gelation was observed by optical microscope for both samples (Figure S2), we were not able to measure the phase transition behavior by rheometer (Figure S3). This is because too large microgel–silica nanoparticle aggregates hinder homogenous and continuous microgel networks, which implies a delicate control of dispersion stability is crucial for engineering the thermogelling behavior of microgel–nanoparticle composites.

## 4. Conclusions

A reverse sol–gel transition behavior of microgel dispersion has long been studied in the biomedical research field. In particular, a p(NiPAm)-based microgel dispersion was widely studied due to a moderate volume phase transition temperature around human body temperature. In this report, we showed the unique phase transition behavior of p(NiPAm)-based microgel dispersion is maintained in the presence of nanoparticle additives. When the p(NiPAm-co-HEMA) microgel was mixed with silica nanoparticles at a 1:1 weight ratio, it showed a stable phase transition behavior. Although some micro aggregation between microgels and silica nanoparticles was observed, a reversible global phase transition behavior was also observed. It is noted that a bulk gel strength was affected by the existence of additives as the jammed colloidal nanoparticle reinforced the microgel networks. The result implies that the microgel system can be potentially feasible for versatile applications that demand a complex colloidal system. For example, one can introduce the conducting colloids to microgel dispersion to provide self-healing properties to electronic materials. By incorporating plasmonic or fluorescent colloids with microgels, optical signals can be amplified or reduced because the sol–gel transition also drives the volume change of the system. Rheological property can also be tuned by gelation, which can be applied for the formulation of a slurry composed of a complex colloidal system. Therefore, we believe that the microgel–colloid composite system can be a strong candidate to be applied for designing a smart and functional colloidal system in the future.

## Figures and Tables

**Table 1:** Reaction recipe for the synthesis of poly(N-isopropylacrylamide-co-2-hydroxyethyl methacrylate) (p(NiPAm-co-HEMA)) microgels.

NiPAm: HEMA Molar Ratio	NiPAm		HEMA		MBA		SDS		APS		Reaction Time
	mmol	g	mmol	g	mmol	g	mmol	g	mmol	g	h
10:0	250	28.290	0.00	0.000	5.00	0.771	1.00	0.288	1.50	0.342	1
9:1	225	25.461	25.0	3.254							1
8:2	200	22.632	50.0	6.507							1
7:3	175	19.803	75.0	9.761							1
6:4	150	16.974	100	13.014							0.75
5:5	75	8.487	75	9.761	3.00	0.463					0.75

## Data Availability

All data in this study are available from the corresponding author upon reasonable request.

## Supplementary Materials

The following are available online at https://www.mdpi.com/1996-1944/14/5/1212/s1, Figure S1: (a) UV-vis spectrum of p(NiPAm-co-HEMA) microgel dispersion with molar ratio between NiPAm and HEMA of 7:3 at various pH values. The increase of transmittance at acidic of pH 4 and alkalic of pH 10 is attributed to the sedimentation of microgels. (b,c) photograph images of p(NiPAm-co-HEMA) microgel dispersion at (b) pH 4, and (c) pH 10. Photos were taken after slightly shaking the sedimented microgels solution for the visualization.; Figure S2: Microscopic images of p(NiPAm-co-HEMA) microgel and silica nanoparticle composite. The composite was 1:5 in weight ratio. (a) Small aggregates at room temperature. (b) local aggregation as the temperature increases. (c) Global aggregation and gelation of the composite with 1:5 weight ratio.; Figure S3: Evolution of dynamic modulus of 1:5 weight ratio composite. Turnover of G′(storage modulus) and G″(loss modulus) was observed 51.3 °C.; Video S1: Supporting Movie S1; Video S2: Supporting Movie S2.
