# Supplementary material for: Thermogelling Behaviors of Aqueous Poly(N-Isopropylacrylamide-co-2-Hydroxyethyl Methacrylate) Microgel–Silica Nanoparticle Composite Dispersions
Source: Materials (Basel). 2021 Mar 4;14(5):1212. doi: 10.3390/ma14051212 (PMC7961736; doi:10.3390/ma14051212)
Supplement: Supplementary file 1 [file materials-14-01212-s001.zip › materials-1104913 SM final.docx]

Supporting Information

Thermogelling Behaviors of Aqueous Poly(N-isopropylacrylamide-co-2-hydroxyethyl methacrylate) Microgel-Silica Nanoparticle Composite Dispersions

**Byung Soo Hwang,^1,†^ Jong Sik Kim^2,†^ , Ju Min Kim^1,2,*^ and Tae Soup Shim^1,2,*^**

^1^ Department of Chemical Engineering and ^2^Department of Energy Systems Research, Ajou University, Suwon 16499, Republic of Korea

***** Correspondence: jumin@ajou.ac.kr; tsshim@ajou.ac.kr

† These authors contributed equally to this work.


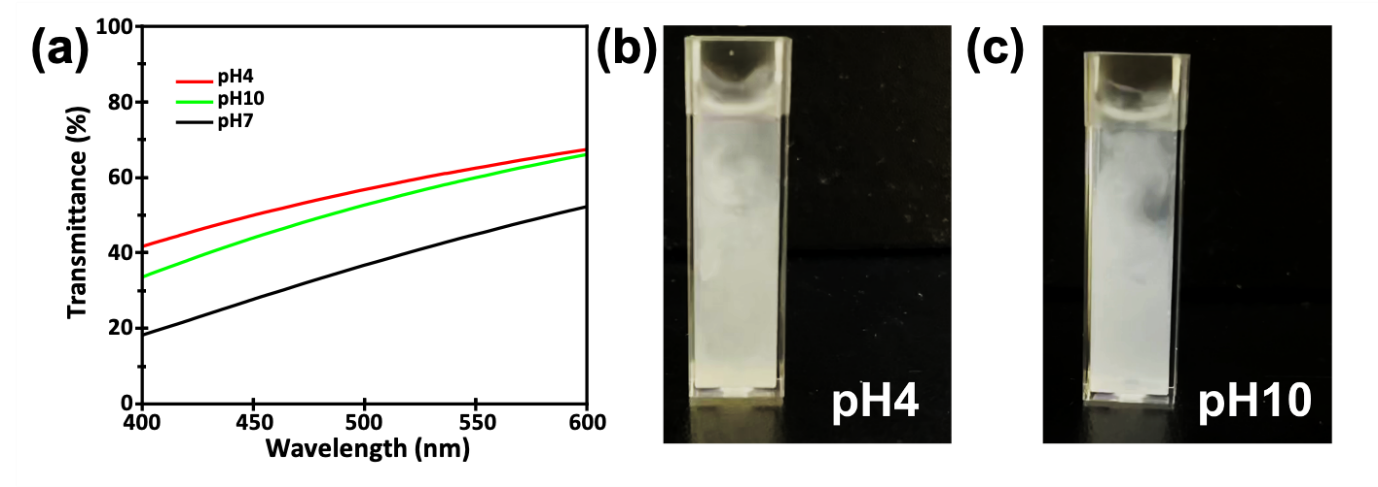


Figure 1. (a) UV-vis spectrum of p(NiPAm-co-HEMA) microgel dispersion with molar ratio between NiPAm and HEMA of 7:3 at various pH values. The increase of transmittance at acidic of pH 4 and alkalic of pH 10 is attributed to the sedimentation of microgels. (b, c) photograph images of p(NiPAm-co-HEMA) microgel dispersion at (b) pH 4, and (c) pH 10. Photos were taken after slightly shaking the sedimented microgels solution for the visualization.


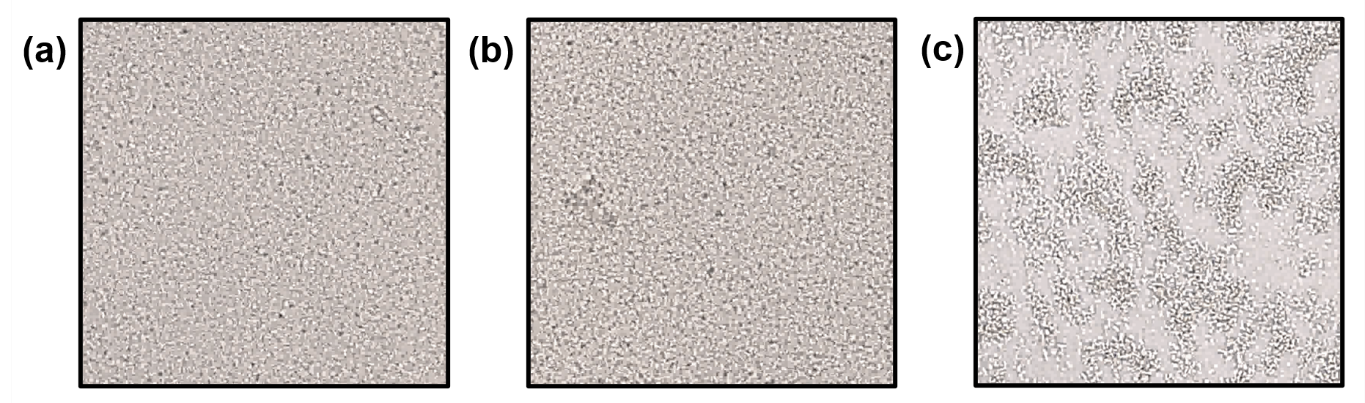


Figure 2. Microscopic images of p(NiPAm-co-HEMA) microgel and silica nanoparticle composite. The composite was 1:5 in weight ratio. (a) Small aggregates at room temperature. (b) local aggregation as the temperature increases. (c) Global aggregation and gelation of the composite with 1:5 weight ratio.


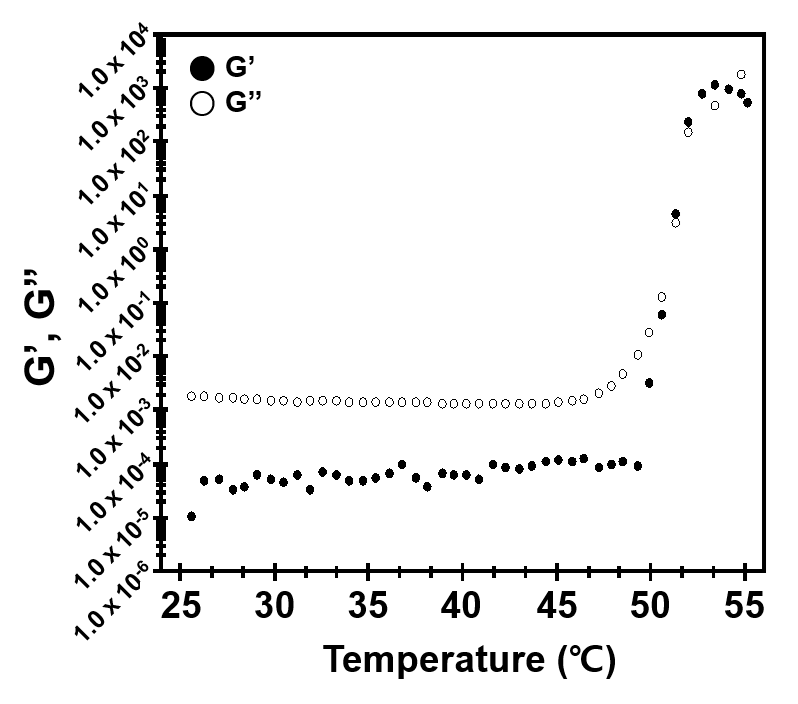


Figure 3. Evolution of dynamic modulus of 1:5 weight ratio composite. Turnover of G’(storage modulus) and G”(loss modulus) was observed 51.3℃. .
